# Supplementary material for: Epigenetic Modifiers: Exploring the Roles of Histone Methyltransferases and Demethylases in Cancer and Neurodegeneration
Source: Biology (Basel). 2024 Dec 3;13(12):1008. doi: 10.3390/biology13121008 (PMC11673268; doi:10.3390/biology13121008)
Supplement: Supplementary file 1 [file biology-13-01008-s001.zip › biology-3298876-supplementary.pdf]

Table S1. HMTs and HDMs Implicated in Cancers and Neurodegenerative Diseases.

	Mark	Gene	Cancer Types	Tumor Suppressor or Oncogene	Neuro-degenerative Diseases	Reference
**HMTs**	H3K4	PRDM9	GBM, pancancer	Oncogene	-	[20,21,43]
		ASH1L	Pancancer	Oncogene	DLB	[28,35,60,61,103]
		SETMAR	GBM, pancancer	CTD	-	[27,29–31]
		SETD1A	Breast, pediatric HGG	Oncogene	-	[22,23]
		SET7/9	Glioma	Tumor Suppressor	-	[26]
		SMYD1	MB	Oncogene	AD, PD, HD	[104,105]
		SMYD2	Glioma, GBM, LGG, pancancer	CTD	-	[38,39]
		KMT2A	GBM, LGG, pancancer	CTD	AD	[24,106,107]
		KMT2C	Pancancer	Tumor Suppressor	-	[3,24]
		KMT2D	MB, Pancancer	Tumor Suppressor	-	[25]

	H3K9	PRDM3	Pancancer	Tumor Suppressor	-	[57]
		PRDM16	Pancancer	Tumor Suppressor	AD	[57]
		SUV39H1	DMG, GBM pancancer	Oncogene	-	[32,33,108]
		SETDB2	Pancancer	Oncogene	AD	[69]
		G9a	GBM, MB, pancancer	Oncogene	AD, PD, HD	[85,88,91,92]
		GLP	MB, ganglioglioma, pancancer	CTD	AD, PD, HD	[85,86,91,92]
		PRDM2	Pancancer	Tumor Suppressor	AD	[65]
	H3K27	PRC2	MB, pancancer	CTD	AD	[109–111]
	H3K36	ASH1L	Pancancer	Oncogene	DLB	[28,61,103]
		SETMAR	GBM, pancancer	CTD	-	[2,29,31]
		NSD1	DMG, pancancer	CTD	-	[34,35,39]
		NSD2	DMG, pancancer	Oncogene	-	[35,36,39]
		NSD3	Pancancer	Oncogene	AD, PD	[35]

		SMYD2	Glioma, GBM, LGG, pancancer	Oncogene	-	[37]
		SETD2	GBM, pediatric HGG, LGG, pancancer	Tumor Suppressor	HD	[28,80,84]
		SETD3	Pancancer	CTD	-	[13,14]
	H3K79	DOT1L	GBM, pancancer	Oncogene	-	[40–42]
	H4K20	SET-8	Pancancer	CTD	-	[112]
		SUV4-20H2	Pancancer	CTD	-	[59]
		PRDM6	MB, pancancer	CTD	-	[43,44,113]
**HDMs**	H3K4	NO66	GBM, Glioma, pancancer	Oncogene	-	[45]
		LSD1	GBM, MB, pancancer	Oncogene	-	[48,49]
		JARID1A	GBM, pancancer	CTD	-	[47]
		JARID1C	Pancancer	Tumor Suppressor	-	[47]

	H3K9	PHF2	Pancancer	Tumor Suppressor	AD, PD	[11,72,73]
		JMJD1A	Pancancer	Oncogene	AD	[67]
		KDM7A	Pancancer	Oncogene	-	[62,64]
		PHF8	Pancancer	Oncogene	PD	[114,115]
	H3K27	UTX	Pancancer	Tumor Suppressor	HD	[56,77]
		JMJD3	Pancancer	Oncogene	AD, PD	[75,76]
		KDM7A	Pancancer	Oncogene	-	[62,64]
		PHF8	Pancancer	Oncogene	PD	[114,115]
	H3K36	NO66	GBM, glioma, pancancer	Oncogene	-	[45]
		JMJD5	GBM, glioma pancancer	CTD	-	[50,51]
		KDM2A	GBM, glioma, pancancer	Oncogene	AD	[93,95,100]
		KDM2B	GBM, glioma, pancancer	Oncogene	AD	[97,98,100]
		JMJD2A	Pediatric HGG, glioma, pancancer	Oncogene	AD	[100,116,117]

		JMJD2C	DMB, pancancer	Oncogene	-	[118,119]
	H4K20	KDM7A<br><br>PHF8	Pancancer<br><br>Pancancer	Oncogene<br><br>Oncogene	-<br><br>PD	[62,64]<br><br>[114,115]

Pancancer, modifier implicated in 3 or more cancer types; CTD; cancer type-dependent; GBM, glioblastoma; HGG, high-grade glioma; DMG, diffuse midline glioma; MB, medulloblastoma; DMB, desmoplastic medulloblastoma; AD, Alzheimer's Disease; PD, Parkinson's Disease; HD, Huntington's Disease; DLB, Dementia with Lewy bodies; -, no research found.

Table S2. HMTs and HDMs Implicated in Both Brain Cancers and Neurodegenerative Diseases.

	Mark	Gene	Brain Cancer	Tumor Suppressor or Oncogene	Neuro-degenerative Diseases	Reference
**HMTs**	H3K4	SMYD1<br><br>KMT2A	MB<br><br>GBM, LGG	Oncogene<br><br>Tumor Suppressor	AD, PD, HD<br><br>AD	[105]<br><br>[24,107,108]
	H3K9	G9a<br><br>GLP	GBM, MB<br><br>MB, ganglioglioma	Oncogene<br><br>CTD	AD, PD, HD<br><br>AD, PD, HD	[89,92,93]<br><br>[87,92,93]
	H3K27	PRC2	MB	CTD	AD	[110–112]
	H3K36	SETD2	GBM, pediatric HGG, LGG	Tumor Suppressor	HD	[81,84,85]
**HDMs**	H3K36	KDM2A	GBM, glioma	Oncogene	AD	[94,96,101]

		KDM2B	GBM, glioma	Oncogene	AD	[99–101]
		JMJD2A	Pediatric HGG, glioma	Oncogene	AD	[101,117,118]

Pancancer, modifier implicated in 3 or more cancer types; CTD, cancer type-dependent; GBM, glioblastoma; HGG, high-grade glioma; MB, medulloblastoma; AD, Alzheimer's Disease; PD, Parkinson's Disease; HD, Huntington's Disease; -, no research found.
